# Noninvasive prenatal testing for assessing foetal sex chromosome aneuploidy: a retrospective study of 45,773 cases

**DOI:** 10.1186/s13039-020-00521-2

**Published:** 2021-01-06

**Authors:** Xinran Lu, Chaohong Wang, Yuxiu Sun, Junxiang Tang, Keting Tong, Jiansheng Zhu

**Affiliations:** 1 Affiliated Maternity and Child Health Hospital of Anhui Medical University, Hefei, China; 2 Maternity and Child Health Hospital of Anhui Province, Hefei, China

**Keywords:** Noninvasive prenatal testing, Sex chromosome aneuploidy, Positive predictive value, Advanced maternal age, High-throughput sequencing, Prenatal screening, Prenatal diagnosis

## Abstract

**Objective:**

To assess the positive predictive value (PPV) of noninvasive prenatal testing (NIPT) as a screening test for sex chromosome aneuploidy (SCA) with different maternal characteristics and prenatal decisions in positive cases.

**Materials and methods:**

We retrospectively analysed 45,773 singleton pregnancies with different characteristics that were subjected to NIPT in the Maternity and Child Health Hospital of Anhui Province. The results were validated by karyotyping. Clinical data, diagnostic results, and data on pregnancy outcomes were collected.

**Results:**

In total, 314 cases were SCA positive by NIPT; among those, 143 underwent invasive prenatal diagnostic testing, and 58 were true-positive. Overall, the PPVs for 45,X, 47,XXX, 47,XXY and 47,XYY were 12.5%, 51.72%, 66.67% and 83.33%, respectively. Interestingly, when only pregnant women of advanced maternal age (AMA) were screened, the PPVs for 45,X, 47,XXX, 47,XXY and 47,XYY were 23.81%, 53.33%, 78.95%, and 66.67%, respectively. The frequency of SCA was significantly higher in the AMA group than in the non-AMA group. The frequencies of 47,XXX and 47,XXY were significantly correlated with maternal age.

**Conclusion:**

NIPT performed better in predicting sex chromosome trisomies than monosomy X, and patients with 45,X positive foetuses were more eager to terminate pregnancy than those with 47,XXX and 47,XYY. AMA may be a risk factor of having a foetus with SCA. Our findings may assist in genetic counselling of AMA pregnant women. Our pre- and posttest counselling are essential for familiarizing pregnant women with the benefits and limitations of NIPT, which may ease their anxiety and enable them to make informed choices for further diagnosis and pregnancy decisions.

**Electronic supplementary material:**

The online version of this article (10.1186/s13039-020-00521-2) contains supplementary material, which is available to authorized users.

## Background

Birth defects are structural deformities or functional abnormalities caused by congenital, genetic, or environmental factors [1]. Chromosomal abnormalities can be divided into chromosomal numerical abnormalities and chromosomal structural abnormalities. Chromosomal numerical abnormalities are usually a result of damage to the mitotic mechanism of cells, while structural abnormalities are a product of chromosome breaks and relocations. The phenotype of chromosomal abnormalities usually includes male infertility, spontaneous abortion, stillbirth, neonatal death, and congenital malformations. Thus, prenatal testing has become critical in preventing congenital disabilities [2].

Sex chromosome aneuploidy (SCA) refers to conditions caused by numerical abnormalities in X and Y chromosomes, such as Turner syndrome (45,X), triple X syndrome (47,XXX), Klinefelter syndrome (47,XXY), and Jacob’s syndrome (47,XYY) [1]. 45,X is a common chromosomal disorder affecting approximately 1 in 2500 to 1 in 2000 of live-born female infants [3], the common clinical manifestations of which include congenital cardiac anomalies, renal anomalies, and acquired metabolic syndrome [3, 4]. 47,XXX occurs in 1 in 1000 female births [5]. In contrast to other trisomies, most of the girls born with triple X chromosomes do not have a characteristic physical appearance at birth, but their heights tend to be variable. In addition, some individuals may exhibit developmental delays (speech and motor), learning or intellectual disability, and psychiatric problems [4, 5]. 47,XXY is the most common SCA, and it occurs in 1 of every 660 males [6]. In adults, the extra X chromosome may affect testicular development, which will result in infertility and hypogonadotropic hypogonadism [6]. Males with 47,XXY also have a higher risk of learning disabilities, developmental delays, cardiometabolic disease, typical physical symptoms and neurodevelopmental manifestations [6, 7]. 47,XYY occurs in 1 in 1000 males [8], and these males tend to have tall stature, face social obstacles, have behavioural problems, and exhibit language impairment. The cognitive phenotype of males with 47,XYY typically includes normal to mildly diminished general intelligence [8].

The phenotypes of SCA patients include a broad range of associated symptoms that vary in severity, depending on the timing of diagnosis and type of SCA [4, 5, 7–9]. The frequency of SCA is estimated to be 1 in every 500 live births. Approximately 75–90% of cases are undiagnosed during the individual’s lifetime [10]. Recently, studies reported that postnatal hormone therapy could have positive effects on behavioural phenotype if applied to SCA patients early. Moreover, screening and prenatal detection of SCA can provide an opportunity for early management, there by improving the quality of life of the affected child.

Before the introduction of noninvasive prenatal testing (NIPT), amniocentesis, chorionic villus sampling, and cord blood collection were the most common tests for assessing sex chromosome abnormalities. However, this method has been associated with an increased risk of procedure-related miscarriage (0.5–1.0%) and maternal anxiety. In 1997, Lo et al. [11]. discovered cell-free DNA derived from the Y chromosome in the maternal plasma of pregnant women carrying male foetuses. NIPT analyses cell-free DNA, which is a mixture of maternal DNA and a low percentage of foetal DNA believed to originate from the trophoblasts, in a pregnant woman’s blood [1, 12]. NIPT uses massively parallel sequencing for prenatal foetal aneuploidy screening. Currently, with the development of high-throughput sequencing technology, NIPT has been highly recommended in clinical practice as a screening method for foetal trisomy 21 (T21), trisomy 18 (T18), and trisomy 13 (T13) among pregnant women with a high risk of abnormal serological screening test results in the second trimester [1, 13]. Extensive studies have demonstrated the high sensitivity and specificity of NIPT for screening T21, T18, and T13, with sensitivity values of 95.9%, 86.5%, and 77.5%, respectively, and specificity values of 99.9%, 99.8%, and 99.9%, respectively [14]. Moreover, studies have reported positive predictive values (PPVs) of 65–94%, 47–85%, and 12–62% for T21, T18, and T13, respectively [15]. In addition, NIPT has the advantage of being noninvasive, thus avoiding the 0.5–1.0% risk of miscarriage associated with amniocentesis/chorionic villus sampling [16, 17].

Since the implementation of the two-child policy in China in 2014, the pregnancy rate among women aged ≥ 35 years has gradually increased [5]. At present, it is generally believed that advanced maternal age (AMA) is an important risk factor for chromosomal abnormalities [2]. An understanding of the correlation between SCA frequency and age would provide a solid basis for the development of appropriate prenatal screening and diagnostic methods.

The aim of this study was to assess the PPV of NIPT as a screening test for SCA with different maternal characteristics and prenatal decisions.

## Results

### Maternal characteristics

A total of 45,773 maternal blood samples from singleton pregnancies were collected in the Maternity and Child Health Hospital of Anhui Province between June 1, 2015, and June 30, 2019. Gestational age at the time of amniocentesis ranged from 12^+0^ to 26^+6^, and maternal age ranged from 16 to 45 years. Before undergoing NIPT, pregnant women were subjected to screening tests, which included serological screening tests and foetal ultrasonography.

In total, there were 16,921 (36.97%) AMA (age ≥ 35 years) cases and 19,820 (43.30%) cases at high or critical risk for developing SCA. B-ultrasound showed that 503 (1.10%) foetuses were soft markers and that 86 (0.19%) foetuses had increased nuchal translucency (NT,  ≥ 3 mm). Moreover, 500 (1.09%) pregnant women missed other screening opportunities, and 7943 (17.35%) pregnant women with no clinical indications underwent NIPT (Table 1).

**Table 1:** Maternal characteristics of pregnant women who underwent NIPT

Maternal age (years)	Cases (%)
< 30	17,449 (38.12%)
30 ≤ year ≤ 34	11,403 (24.91%)
35 ≤ year ≤ 39	14,353 (31.36%)
> 39	2568 (5.61%)
*Gestational age at NIPT (weeks)*	
12–15	4853 (10.60%)
16–19	33,066 (72.24%)
20–23	6740 (14.72%)
24–26	1037 (2.27%)
> 26	77 (0.17%)
*Clinical features*	
Advanced maternal age (age ≥ 35 years)	16,921 (36.97%)
High or critical risk of serological screening	19,820 (43.30%)
Foetal soft markers by B-ultrasound	503 (1.10%)
Increased NT	86 (0.19%)
Missed other screening opportunities	500 (1.09%)
No clinical indications	7943 (17.35%)

### The clinical value of NIPT for screening for foetal SCA and pregnancy outcome

As shown in Table 2, 143 pregnant women (45.54%) accepted the prenatal diagnosis. All patients received the results within 3 weeks. After prenatal diagnosis, 58 pregnant women were true-positive. One hundred forty-seven pregnancies were positive for 45,X. Of them, 56 pregnancies underwent invasive prenatal testing (38.10%). Seven cases were true-positive, with a 12.50% PPV for NIPT. Six patients with SCA terminated their pregnancies with a termination rate of 85.71%. Two of them due to foetuses structurally abnormal at ultrasound. Among patients with 47,XXX, karyotype information was available for 29 out of 61 NIPT patients with results (47.54%). Among them, 15 cases were true-positive, with a PPV of 51.72%. The NIPT and karyotype analysis were fully concordant for 15 patients, of whom 3 decided to terminate their pregnancies (20%). Among patients with 47,XXY, karyotype information was available for 39 out of 71 NIPT results with a prenatal diagnosis (54.93%). Twenty-six cases were true-positive, with a PPV of 66.67%. Nineteen pregnant women decided to terminate their pregnancies (73.08%). Karyotype information for patients with 47,XYY was available for 12 out of 35 NIPT patients with results with a prenatal diagnosis (34.29%). Ten cases were confirmed to be true-positive, with a PPV of 83.33%. Of these ten patients, one decided to terminate their pregnancies (10%).

**Table 2:** The results of prenatal diagnosis and clinical outcome

SCA type	NIPT positive (N)	Karyotype analysis			Unverified (N)	Termination rate (%)
		True positive (N)	False positive (N)	PPV (%)		
45,X	147	7	49	12.5	91	85.71
47,XXX	61	15	14	51.72	33	20
47,XXY	71	26	13	66.67	32	73.08
47,XYY	35	10	2	83.33	22	10

SCA, sex chromosome aneuploidy; PPV, positive predictive value

Moreover, the proportions of sex chromosome trisomy and monosomy were highly different. Among 167 cases with positive screening results for sex chromosome trisomy, 51 out of 80 NIPT results were confirmed to be true-positive by invasive prenatal testing (63.75%). Among 56 out of 147 cases of sex chromosome monosomy with a prenatal diagnosis, 7 cases were consistent with the NIPT results (12.50%). Chi square tests were used to further examine the associations between sex chromosome trisomy and monosomy X. Statistical significance was observed between the two groups (χ^2^ = 35.374, *P* < 0.05).

After prenatal diagnosis, all patients received prenatal genetic counselling. Among the 85 patients with false-positive results, 67 were successfully followed up. They all successfully delivered their babies. A total of 171 pregnant women refused prenatal diagnosis, and 126 had effective follow-up results. Moreover, 86 pregnant women refused prenatal diagnosis due to abortion-related anxiety, and 33 pregnant women refused prenatal diagnosis due to B-ultrasound results showing a severe foetal condition. Another 7 pregnant women experienced spontaneous abortion or stillbirth during gestation.

The PPV of SCA detected by NIPT was 40.56% (58/143), and the PPV of SCA in pregnant women in the AMA group (≥ 35 years) was 50.79% (32/63). The PPVs were 30.43% (14/46) in the high or critical risk group, 40% (10/25) in the no clinical indications group, 25% (1/4) in the missed other screening opportunities group, and 33.33% (1/3) in the increased NT group.

A total of 85 false-positive NIPT cases were identified. Importantly, 32 cases were suspicious for an abnormal maternal ChrX karyotype, detected by NIPT, including 23 cases with an abnormal ChrX gain and 9 cases with an abnormal ChrX loss. Based on genetic counselling, 32 cases underwent maternal peripheral blood karyotyping. Moreover, 2 cases among 23 cases with an abnormal ChrX gain were diagnosed with 47,XXX, while 2 cases among 9 cases with an abnormal ChrX loss were diagnosed with mosaicism 45,X/47,XXX and mosaicism 47,XXX/45,X/46,XY.

In our study, a pregnant woman was referred for NIPT at 16 weeks gestation because of advanced maternal age, which turned to be a positive result for 45,X. The woman decided to undertake amniocentesis at 17^+6^ weeks, and a normal result was identified. G-banded karyotyping analysis was also carried out on peripheral blood lymphocytes derived from the couple and ruled out abnormal chromosome karyotype. Then with normal ultrasound, the women chose to continue pregnancy. The case delivered live birth at 39 weeks, and foetal and placental tissue was retained for measurement of suspected SCA mosaicism by DNA sequencing. Placental biopsies taken at the center of the tissue and at the middle and edge of the maternal–fetal interface, had variable 45,X mosaicism levels of 70%, 50%, and 70%, respectively. The examination of the placental tissue at three sites showed 45,X mosaicism.

### Relationship between SCA frequency and maternal age

We evaluated the relationships between different PPVs and maternal age. For SCA, the PPV increased with maternal age, and was the highest among women aged > 39 years. In our study, there were 16,921 (36.97%) patients with AMA (≥ 35 years), accounting for a relatively large population. The PPV, which was 50.79% for SCA in AMA women, was slightly higher than that of all samples (40.56%). The frequency of SCA (32/16872) in the AMA group was significantly higher than in the non-AMA group (26/28730) (χ^2^ = 8.229, *P* = 0.004, OR = 2.098, 95% CI-1.250 to 3.521) (Table 3). The frequencies of 45,X and 47,XYY did not differ among age groups of pregnant women (χ^2^ = 4.328, *P* > 0.05 for 45,X, and χ^2^ = 1.074, *P* > 0.05 for 47,XYY). However, the frequencies of 47,XXX and 47,XXY were different among the different age groups of (χ^2^ = 12.616, *P* < 0.05 for 47,XXX, and χ^2^ = 12.570, *P* < 0.05 for 47,XXY) (Table 4).

**Table 3:** The NIPT results of SCA between AMA and non-AMA groups

Age	SCA	Non-SCA	OR (95% CI)	*P* value
AMA	32	16,840	2.098 (1.250-3.521)	0.004
Non-AMA	26	28,704

**Table 4:** NIPT results for SCA screening in pregnant women of different age groups

NIPT results	All cases			AMA cases			Age < 30 years			30 ≤ age ≤ 34 years			35 ≤ age ≤ 39 years			Age > 39 years		
	TP	FP	PPV	TP	FP	PPV	TP	FP	PPV	TP	FP	PPV	TP	FP	PPV	TP	FP	PPV
45,X	7	49	12.5	5	16	23.81	2	18	10	0	15	0	4	14	22.22	1	2	33.33
47,XXX	15	14	51.72	8	7	53.33	4	4	50	3	3	50	4	5	44.44	4	2	66.67
47,XXY	26	13	66.67	15	4	78.95	4	6	40	7	3	70	10	4	71.43	5	0	100
47,XYY	10	2	83.33	4	2	66.67	3	0	100	3	0	100	4	2	66.67	/	/	/
SCA suspected abnormal	0	7		0	2		0	2		0	3		0	2		/	/	/
Total	58	85	40.56	32	31	50.79	13	30	30.23	13	24	35.14	22	27	44.9	10	4	71.43

NIPT, noninvasive prenatal testing; SCA, sex chromosome aneuploidy; AMA, advanced maternal age; TP, true positive; FP, false positive; PPV, positive predictive value

## Discussion

Several recent studies reported the sensitivity, specificity, and PPV of NIPT for T21, T18, and T13 screening. However, to date, no large-scale clinical studies have been conducted to assess the efficiency of NIPT for detecting SCA. To the best of our knowledge, this is the largest study (45,773 cases) to evaluate NIPT for SCA. According to our data, the overall PPV of SCA detected by NIPT was 40.56%, within the range of PPV for SCA screening between 30 and 60% [12, 13, 15, 18, 19]. When categorized by individual SCAs, the PPVs were 12.5% for 45,X, 51.72% for 47,XXX, 66.67% for 47,XXY, and 83.33% for 47,XYY, which were similar to those reported by other studies [1, 13, 20]. However, in our study, we noticed that different pregnancy characteristics showed different PPVs; for example, the PPV of SCA in AMA pregnancy cases (50.79%) was higher than that in all cases (40.56%). Moreover, the overall termination rate was 50% for SCA (when mosaic cases were included) and 41.38% (when mosaic cases were not included); the termination rates for foetal SCA were 85.71% for 45,X, 20% for 47,XXX, 73.08% for 47,XXY, and 10% for 47,XYY [13].

Our study showed that NIPT performed better in predicting sex chromosome trisomies than monosomy X. This may be due to the following. [1] There are 1098 genes on the X chromosome and 78 genes on the Y chromosome, and 58 genes are homologous genes on both sex chromosomes. The majority of the genes (29 genes) are at the ends of the sex chromosomes. [2] The low guanosine-cytosine content of the X chromosome leads to highly variable amplification of the X chromosome. [3] The nonrandom inactivation of the X chromosome in placental tissue might be the reason for the low PPV of Turner syndrome, with the paternal X chromosome tending to be inactivated in XX female trophoblasts [1, 13]. In addition, it was reported that there is an age-related X chromosome loss in normal female white blood cells, which may influence the effectiveness of predicting foetal 45,X [13], although this was not observed in our study.

The PPV of SCA is lower than that of other common chromosome aneuploidies. The reason is that sex chromosome abnormalities are less prevalent [15]. Wang et al. reported that 8.6% of positive results for SCA were due to maternal mosaicism [21], which was later confirmed by other studies [1, 13, 22]. With the combination of NIPT and maternal peripheral blood karyotype analysis, we found that approximately 12.5% of the discordant NIPT SCA results were due to maternal mosaicism in our study. Previous studies have demonstrated that the identification of maternal karyotypes tends to decrease the rate of false-positive SCA and can offer an explanation for the false-positive results for SCA [13, 14, 23]. With regard to the discordance between NIPT and invasive prenatal testing, another reason is confined placental mosaicism (CPM), which occurs in approximately 1–2% of all pregnancies [12]. The origin of most cell-free foetal DNA (cffDNA) in the maternal plasma is mostly the apoptosis of placental cells from the cytotrophoblast [24]. The mosaicism degree reduces the effective cffDNA concentration in maternal plasma, thus affecting the performance of NIPT in detecting foetal aneuploidies. Grati et al. determined the potential contribution of CPM to the NIPT false‐positive rate, demonstrating that chromosomes 13 and X were more likely to be associated with CPM than chromosomes 18 and 21 [25]. In current study, postnatal placental analysis was preformed in one case with CPM. This case illustrates that extensive cytogenetic analysis can be required to identify CPM, and that patients should be counseled regarding the possibility of discordant NIPT results. However, obtaining the placenta for clinical cases remains challenge for clinical laboratories preforming analysis of discordant NIPT results. Our results verified the value of determining the maternal karyotype and examination of placental tissue in increasing the accuracy of reporting NIPT results for chromosomes X and Y; however, further studies are needed to provide more clinical data in support of this premise.

Despite the prenatal diagnosis of foetal SCA, some pregnant women were willing to continue their pregnancies, and there were differences in the rates of pregnancy termination between different types of SCAs. Pregnant women whose foetuses were 45,X or 47,XXY were more eager to terminate their pregnancies than those whose foetuses were 47,XXX or 47,XYY, which was consistent with other studies [13, 26]. Gruchy et al. [27] reported that with regard to Turner syndrome, the rate of pregnancy termination was closely related to the 45,X karyotype, mosaic karyotype, and structural abnormalities of the X chromosome. In our study, pregnant women whose foetuses were 45,X with mosaicism of 23.08% had a stronger tendency to continue their pregnancies, while those with mosaicism of 28.38%, 48.53%, 59.38%, 76.67%, and 80.49% were more inclined to terminate their pregnancies. Current studies report that almost all pregnant women carrying foetuses with 45,X decide to terminate their pregnancy [28]. To some extent, the parental decisions for pregnancy termination may have been related to the types of SCA, level of prenatal genetic counselling, history of infertility, parental and social acceptance, economic condition, and related factors.

In clinical practice, AMA pregnant women are willing to accept NIPT as a noninvasive and accurate screening method. In this study, the proportion of AMA pregnant women was the second highest in the NIPT screening population, reaching 36.97%. Among them, NIPT detected 108 cases of SCA; 63 cases underwent amniocentesis, and 32 (50.79%) cases were confirmed to be true-positive. Of all the confirmed SCA cases, 55.17% (32/58) were AMA pregnant women. The study showed that NIPT could be used as a useful screening test for SCA in AMA pregnant women, which was similar to the results reported by Zheng et al. [29]. The differences in the frequency of SCA were statistically significant among the age groups, and the frequency was significantly higher in the > 39 years age group (*P* < 0.05). And the frequency of SCA in the AMA group was significantly higher than in the non-AMA group. We may conclude that AMA may be a risk factor for SCA. Therefore, genetic counselling, combined with serological screening tests and B-ultrasound detection of abnormalities, should be fully carried out for AMA pregnant women. The frequencies of 47,XXX and 47,XXY were significantly correlated with maternal age, whereas the frequencies of 45,X and 47,XYY did not show significant correlations. Although there was no statistical significance regarding the frequency of 47,XYY and maternal age, the frequency of 47,XYY decreased with maternal age in the advanced aged group. To determine whether there is a correlation between maternal age and the frequency of a foetus with 47,XYY, further studies with larger sample sizes are warranted [2]. The risk of 47,XXX increased with AMA, which was consistent with the findings by Zhu et al. [2]. Previous studies showed that for 45,X syndrome, the maternal age coefficient is negative, implying a decreasing frequency among older mothers [2]. However, this was not consistent with our results. Understanding the frequency of SCA has proven valuable in counselling couples who seek advice about the risk of foetal sex chromosome abnormalities with AMA and are considering the option of prenatal diagnosis and termination of pregnancy.

This study has a few limitations. First, the sensitivity, specificity, and negative predictive value were not calculated. New-borns with SCA usually appear phenotypically normal. Therefore, it was difficult to confirm the results of SCA without karyotype analysis during the neonatal period. Second, the number of SCA cases in our study was not large enough to discuss its frequency across different age groups. Therefore, more pregnancies must be evaluated to further understand the association between maternal age and foetal SCA.

Recent studies have demonstrated that early interventions such as postnatal hormone therapy, physical therapy, and occupational therapy could have positive effects on the behavioural phenotype or neurodevelopmental outcomes if applied earlier to SCA patients [9, 30]. Prenatal screening and diagnosis of SCA can provide the opportunity for early intervention, comprehensive postnatal management, and improve the quality of life of the affected child [10]. Sex chromosome abnormalities are more common than major trisomies at birth, and neonates are often phenotypically normal [31]. Conventional prenatal screening cannot be used to directly identify sex chromosome abnormalities that can only be identified using postnatal karyotyping, which in turn may delay the treatment of SCA patients. NIPT allows prenatal screening of SCA. The application of NIPT can allow pregnant women to have an alternate option to invasive prenatal testing for the identification of foetal sex chromosome abnormalities. However, there are still some issues that require further consideration. Due to the existing false-positive rate of NIPT screening for SCA, the number of unnecessary invasive prenatal diagnoses may increase, especially for 45,X [13]. Nevertheless, the benefit of detection for foetal SCA outweighs the risk related to invasive procedures. Some pregnant women may decide to terminate their pregnancies if chromosomal abnormalities are incidentally discovered by SCA screening, which involves ethical issues regarding the mild phenotype of the SCA and the potential increase in the rate of sex selection [13].

With the development of sequencing technology, recent methods have higher accuracy than earlier methods which used to rely on chromosome dosage changes only. Huang et al. [32] presented a novel silicon-based nanostructured microfluidic platform (Cell Reveal™) for capturing circulating fetal nucleated red blood cells (fnRBC) and extravillous cytotrophoblasts (EVT) for cell-based noninvasive prenatal diagnosis (cbNIPD). This method used a microfluidic device coated with specific antibodies to capture the related antigen covered cells. Through the method, the foetal cells could be isolated from the maternal circulation and foetal DNA could be used efficiently to detect copy number abnormalities [32]. In addition, another method, named cell-based noninvasive prenatal testing, could be used to detect the subchromosomal abnormalities (≥ 1 Mb) in foetal cells by using low-coverage shotgun next-generation sequencing [33], which was an ideal way to balance the depth and accuracy of sequencing. Moreover, whole-genome NGS methylomic analysis was also a method to provide a version of the placental methylome from the maternal plasma that could be effective for chromosomal abnormalities testing [34]. All together, NIPT’s research on chromosomal abnormalities has been expanding and deepening, not only staying on the chromosome dosage changes.

## Conclusions

Our data suggest that NIPT can be used to identify foetal SCA by analysing cffDNA obtained from maternal plasma using massively parallel sequencing technology. We found that patients with 45,X foetuses were more eager to terminate their pregnancies. Different maternal characteristics have different PPVs. AMA may be a risk factor for SCA. Additionally, the frequencies of 47,XXX and 47,XXY were correlated with maternal age. Pre- and posttest counselling are essential to familiarizing women with the benefits and limitations of NIPT, which may ease their anxiety and enable them to make an informed choice for further diagnosis and pregnancy decisions.

## Materials and methods

### Subjects

In this study, 45,773 women with singleton pregnancies who underwent NIPT at the Maternity and Child Health Hospital of Anhui Province between June 1, 2015, and June 30, 2019, were recruited. Maternal age, serological screening results, NT, and results were recorded. The inclusion criteria were as follows: [1] gestational week between 12^+0^~26^+6^ and [2] singleton pregnancy. The exclusion criteria were as follows: [1] gestational age < 12 weeks; [2] multiple pregnancies; [3] definite chromosomal abnormalities; [4] pregnant women who underwent an allogeneic blood transfusion, stem cell therapy, transplant surgery, or other procedure; [5] a family history of genetic disease or an indication for a high risk of genetic disease in the foetus; [6] pregnant women with malignant tumours; and [7] other conditions that might affect the accuracy of the results.

The study was approved by the Ethics Committee of Anhui Medical University. Informed consent was obtained from all patients. Before testing, all patients received a consultation with a genetic counsellor or clinical geneticist, after which a prenatal diagnosis was recommended.

### Maternal serum screening tests and ultrasonography

Maternal age ≥ 35 years was defined as AMA [2]. We used a combination of the first trimester screening (from 9 weeks to 13^+6^ weeks) and the second trimester screening (from 15 weeks to 20^+6^ weeks). The serological screening tests used were pregnancy-associated plasma protein A (PAPP-A) and free β-HCG for the first trimester screening and AFP, free β-HCG, and free E3 for the second trimester screening. A time-resolved immunofluorescence assay was used for detection. The risk values were calculated by Lifecycle software (4.0): high risk, T21 > 1/300, T18 > 1/350; critical risk, T21 = from 1/300 to 1/1000, T18 = from 1/350 to 1/1000. The following signs were mainly used as B-ultrasound soft markers: absent or shortened nasal bone, thickened nuchal fold, single umbilical artery, echogenic bowel, pyelectasis, and choroid plexus cysts [35, 36]. NT was measured by a trained sonographer following the Foetal Medicine Foundation protocol, and NT ≥ 3 mm was defined as increased NT [37].

### Noninvasive prenatal testing (NIPT)

A total of 10 mL of maternal peripheral blood was collected in a Cell-Free DNA BCTTM tube (EDTA). Plasma was separated from the maternal plasma by two rounds of centrifugation within 48 h. Whole blood was centrifuged at 1600 × g for 10 min at 4 °C, after which the supernatant was centrifuged at 16000 × g for 10 min. The maternal plasma was immediately stored at − 80 °C until DNA extraction. Extraction of cffDNA, library construction, quality control, and pooling were performed in the laboratory of Maternity and Child Health Hospital of Anhui Province. DNA was extracted by the QIAamp Circulating Nucleic Acid Kit (Qiagen). The pooled library was sequenced using an Illumina with the Data Analysis System for bioinformatic analysis of the sequencing data. The sequencing reads were filtered and aligned to the human reference genome (hg19) [15]. Calculating a Z score per chromosome and chromosome with an absolute value of Z score > 3 was identified with chromosome aneuploidies or microdeletions/microduplications [38]. Additionally, samples with a normalized chromosome value of 3.0 or less were marked as normal.

### Karyotype analysis of amniotic fluid

Karyotype analysis of amniotic fluid was performed for samples collected from pregnant women who were positive for SCA by NIPT and who agreed to undergo invasive prenatal diagnosis, which is the gold standard for chromosome aneuploidy testing. Briefly, under B-ultrasound guidance, 20 mL of amniotic fluid was aseptically withdrawn using amniocentesis. Inoculation, culture, G-banding, and karyotype scanning using a GSL-120 automatic karyotype scanner were then performed. The karyotype was described according to the “International System for Human Cytogenetic Nomenclature, ISCN2016” guidelines. According to the principle outlined by the American College of Medical Genetics and Genomics, 2018, a total of 30 dividing phases were counted per sample using an AI chromosome image analysis system (CytoVision, Switzerland). Five karyotypes were analysed, and double counts were obtained in the case of chimaeras.

## Statistical analysis

Statistical analysis was performed using SPSS version 23 (IBM Corp., Armonk, NY, USA). A Chi square test was applied to compare the frequencies of SCA among pregnant women in different age groups. A *P* value of < 0.05 was considered to be statistically significant.

## Supplementary information

**Additional file 1: Supplementary materials**

## Data Availability

The datasets used and/or analysed during the current study are available from the corresponding author on reasonable request.
