# Supplementary material for: Noninvasive prenatal testing for assessing foetal sex chromosome aneuploidy: a retrospective study of 45,773 cases
Source: Mol Cytogenet. 2021 Jan 6;14:1. doi: 10.1186/s13039-020-00521-2 (PMC7786464; doi:10.1186/s13039-020-00521-2)
Supplement: Supplementary file 1 — Additional file 1: Supplementary materials [file 13039_2020_521_MOESM1_ESM.docx]

**Supplementary material**

**Supplementary Table 1**. The NIPT results of SCA between AMA and non-AMA groups.

|  | SCA | Non-SCA | Total  (N) | Frequency  (‰) |
| --- | --- | --- | --- | --- |
| AMA | 32 | 16840 | 16872 | 1.90 |
| Non-AMA  Total | 26  58 | 28704  45544 | 28730  45602 | 0.90  1.27 |

**Supplementary Table 2**. Maternal age of total and unverified pregnant women who underwent NIPT.

| Total maternal age (years) | Cases |
| --- | --- |
| <30 | 17449 |
| 30≤year≤34 | 11403 |
| 35≤year≤39 | 14353 |
| >39 | 2568 |
| Unverified maternal age (years) |  |
| <30 | 78 |
| 30≤year≤34 | 44 |
| 35≤year≤39 | 40 |
| >39 | 9 |

**Supplementary Table 3**. NIPT results for SCA screening in pregnant women of different age groups.

| NIPT results | All cases (N) | AMA cases (N) | Age<30 years | 30≤age≤34 years | 35≤age≤39 years | Age>39 years |
| --- | --- | --- | --- | --- | --- | --- |
| 45,X | 7 | 5 | 2 | 0 | 4 | 1 |
| 47,XXX | 15 | 8 | 4 | 3 | 4 | 4 |
| 47,XXY | 26 | 15 | 4 | 7 | 10 | 5 |
| 47,XYY | 10 | 4 | 3 | 3 | 4 | / |

**Supplementary Table 4**. Maternal age of unverified pregnant women who underwent NIPT.

| Unverified maternal age (years) | Cases |
| --- | --- |
| <30 | 78 |
| 30≤year≤34 | 44 |
| 35≤year≤39 | 40 |
| >39 | 9 |

**Supplementary Table 5.** The results between NIPT performed in predicting sex chromosome trisomies and monosomy.

|  | SCA | Non-SCA | Total | Detection rate |
| --- | --- | --- | --- | --- |
| Sex chromosome trisomies | 51 | 28 | 80 | 63.75 |
| Sex chromosome monosomy | 7 | 49 | 56 | 12.5 |
| Total | 58 | 77 | 136 | 42.65 |
